# Type VI secretion system effectors as modulators of host immunometabolism: organelle stress, nutritional immunity, and therapeutic opportunities

**DOI:** 10.3389/fmicb.2026.1951748

**Published:** 2026-09-09

**Authors:** Zhenzhen Zhang, Zhen Hou

**Affiliations:** 1 Department of Respiratory Medicine, The People’s Hospital of Weifang, Weifang, China; 2 Department of Anorectal Surgery, Weifang Hospital of Traditional Chinese Medicine, Weifang, China

**Keywords:** endoplasmic reticulum stress, host-directed therapy, immunometabolism, metabolic reprogramming, mitochondrial dynamics, nutritional immunity, redox homeostasis, type VI secretion system

## Abstract

Metabolic reprogramming is a central determinant of host defense and pathogen persistence during infection. Although the bacterial type VI secretion system (T6SS) is primarily recognized as a contact-dependent apparatus for interbacterial competition and effector delivery, emerging evidence indicates that T6SS activity can also influence host metabolism at cellular, nutritional, and microbial-community levels. Here, we organize current evidence into three mechanistic tiers: direct biochemical interference with lipids, metabolites, or metal ions; organelle- and signaling-mediated immunometabolic reprogramming; and indirect metabolic effects arising from T6SS-dependent remodeling of microbial communities. T6SS effectors can disrupt endoplasmic-reticulum lipid homeostasis, activate the unfolded protein response and autophagy, alter mitochondrial Ca2+ handling and dynamics, promote redox imbalance, and modulate metabolically sensitive immune pathways including phosphoinositide 3-kinase (PI3K)-Akt, inflammasome, and cyclic GMP-AMP synthase (cGAS)-stimulator of interferon genes (STING) signaling. T6SS-associated proteins also mediate manganese and zinc acquisition or sequestration, linking microbial nutrient acquisition to host nutritional immunity. At the community level, T6SS-mediated competition may reshape resource allocation, horizontal gene transfer, and microbiome-derived metabolite production. However, while T6SS-induced organelle stress is well established, direct causal links to systemic metabolic diseases, including type 2 diabetes and dyslipidemia, remain unproven. We therefore distinguish direct metabolic measurements from inferences based on organelle damage or signaling changes and discuss strategies to define T6SS-driven metabolic fluxes and evaluate host-directed, anti-virulence, and microbiome-engineering approaches. Viewing the T6SS through an immunometabolic framework may reveal therapeutic vulnerabilities overlooked by conventional models of bacterial toxicity and competition.

## 1 Introduction

Infection is accompanied by extensive metabolic competition and metabolic signaling. Host cells redistribute glucose, amino acids, lipids, and trace metals to support antimicrobial functions, while pathogens adapt their own metabolism and deploy virulence factors to redirect host pathways in favor of survival. These changes are not passive consequences of cellular injury. They determine macrophage activation, inflammatory signaling, organelle function, tissue tolerance, and the capacity of microbial communities to resist invasion. The emerging field of immunometabolism therefore provides a useful framework for connecting molecular virulence mechanisms with disease severity and therapeutic response (Yuan et al., 2025).

The type VI secretion system (T6SS) is a contractile secretion apparatus widely distributed among Gram-negative bacteria. It is best known for delivering toxic proteins into neighboring bacteria, thereby promoting niche occupation, nutrient access, and horizontal gene transfer. T6SSs can also deliver effectors into mammalian cells, fungi, protozoa, and plants, and several recent studies have linked these activities to organelle injury, innate immune signaling, and microbial-community remodeling (Allsopp and Bernal, 2023; Lin et al., 2021; Monjarás Feria and Valvano, 2020; Sana et al., 2017). Nevertheless, most reviews continue to organize T6SS biology around target kingdoms or effector enzymatic classes. This approach is useful for cataloguing diversity but can obscure a second question: how does T6SS activity change the metabolic state of the host and its associated microbiota?

A metabolic interpretation is particularly relevant because many established T6SS targets are themselves metabolic hubs. The endoplasmic reticulum (ER) coordinates lipid synthesis, protein folding, calcium storage, and organelle contact sites. Mitochondria regulate ATP production, redox balance, cell death, and innate immune signaling. Metal ions such as manganese and zinc act both as nutrients and as cofactors for immune enzymes. In parallel, T6SS-mediated killing of microbial competitors changes nutrient availability, metabolite exchange, and the genetic composition of local communities. These processes place the T6SS at the intersection of cellular immunometabolism, nutritional immunity, and microbial ecology.

**FIGURE 1:**
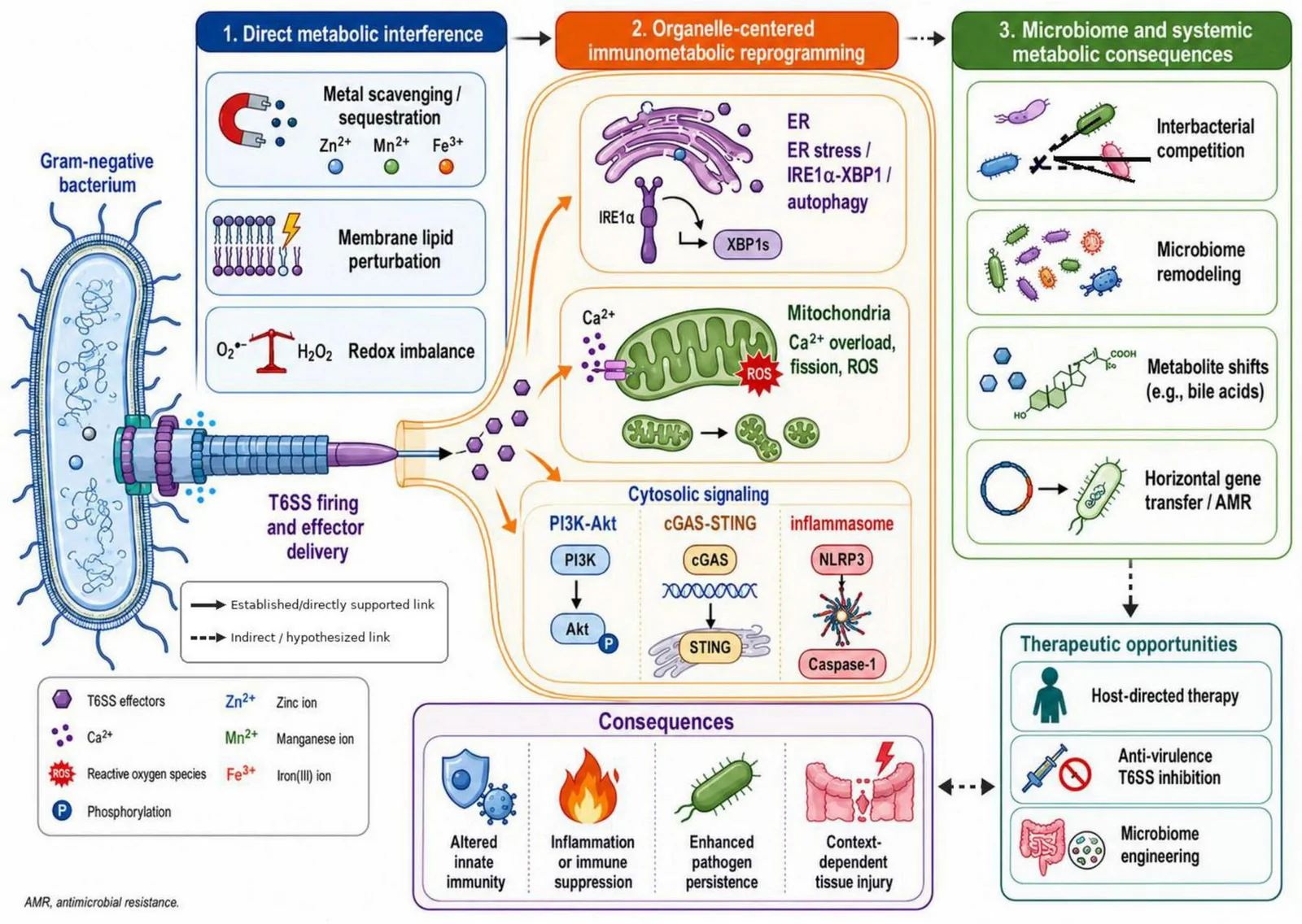
A multilevel framework for T6SS-driven host immunometabolic reprogramming. The type VI secretion system (T6SS) of Gram-negative bacteria delivers effector proteins into host cells, competing microorganisms, or the extracellular environment, thereby influencing metabolism across multiple biological scales. At the first level, T6SS-associated effectors directly interfere with metabolic homeostasis through metal scavenging or sequestration, membrane lipid perturbation, redox imbalance, and evasion of host nutritional immunity. At the second level, intracellular effectors target metabolically active organelles and signaling pathways. Endoplasmic reticulum damage can activate the IRE1α–XBP1 unfolded protein response and autophagy, whereas altered endoplasmic reticulum–mitochondrial Ca^2+^ transfer may promote mitochondrial Ca^2+^ overload, mitochondrial fission, and reactive oxygen species production. T6SS effectors can also modulate metabolically sensitive immune signaling pathways, including PI3K–Akt, cGAS–STING, and inflammasome signaling. At the third level, T6SS-mediated interbacterial competition can remodel microbial community composition, nutrient allocation, metabolite production, and horizontal gene transfer, potentially influencing microbiome-derived metabolites and antimicrobial resistance. Collectively, these effects may alter innate immune responses, promote either inflammation or immune suppression, enhance pathogen persistence, and contribute to context-dependent tissue injury. Potential therapeutic strategies include host-directed modulation of metabolic and stress-response pathways, pathogen-directed inhibition of T6SS activity, and microbiome engineering. Solid arrows denote experimentally established or directly supported links, whereas dashed arrows denote indirect, hypothesized, or system-level relationships pending causal validation. A key within the figure illustrates this convention. AMR, antimicrobial resistance; ER, endoplasmic reticulum; ROS, reactive oxygen species; T6SS, type VI secretion system.

This review evaluates the T6SS as a multiscale regulator of metabolism during host-pathogen interactions. Consistent with the three mechanistic tiers defined below, we focus on three interconnected levels of T6SS action: direct biochemical interference with host lipids, metabolites, and metal ions; organelle- and signaling-mediated immunometabolic reprogramming, including metabolically sensitive innate immune checkpoints; and community-level effects on nutrient competition and microbiome-derived metabolites (Figure 1). We also distinguish direct metabolic evidence from inference based on signaling or morphology, identify major gaps concerning diabetes, obesity, and aging, and discuss opportunities for host-directed therapies (HDTs), anti-virulence strategies, and precision microbiome engineering. Unlike previous reviews that have separately catalogued T6SS structure and effector diversity, microbiome modulation, T6SS-mediated pathogenesis, or nutritional competition (Allsopp and Bernal, 2023; Lin et al., 2021; Monjarás Feria and Valvano, 2020; Sana et al., 2017), this review integrates nutritional immunity and organelle stress with T6SS biology within a single immunometabolic framework.

## 2 Literature search and evidence-appraisal approach

This article is a narrative review rather than a systematic review, and the formal systematic-review procedures specified by PRISMA 2020 (Preferred Reporting Items for Systematic Reviews and Meta-Analyses)–protocol registration, fully reproducible search strings, dual independent screening, and a flow diagram–were not applied (Page et al., 2021); instead, we transparently report the search and evidence-appraisal approach below. PubMed/MEDLINE, Web of Science, and Scopus were searched from database inception to August 2026, without language restrictions, using combinations of the following terms and their variants: “type VI secretion system” or “T6SS,” combined with “effector,” “host metabolism,” “immunometabolism,” “metabolic reprogramming,” “mitochondria,” “endoplasmic reticulum,” “nutritional immunity,” “metal homeostasis,” “manganese,” “zinc,” “inflammasome,” “cGAS-STING,” “microbiome,” “horizontal gene transfer,” “antifungal,” “diabetes,” “obesity,” “aging,” and “host-directed therapy.” The reference lists of retrieved articles and of relevant reviews were screened manually for additional records, and the reference list was updated iteratively during manuscript preparation and revision.

Records were eligible for inclusion if they reported experimental or clinical evidence on T6SS structure, effectors, or associated cargo proteins in relation to host-cell biology, metabolism, innate immunity, metal homeostasis, or microbial-community dynamics. Exclusion criteria were: (i) studies without direct relevance to T6SS biology; (ii) conference abstracts and non-retrievable full texts; and (iii) duplicate reports of the same data set. Because this review makes comparative statements about the strength of evidence (for example, “the strongest current evidence”), each included study was classified according to the three-tier evidence framework defined in section “3.2 Three levels of evidence for T6SS-associated metabolic reprogramming,” and comparative statements in the text were restricted accordingly. Evidence gaps were identified where direct metabolic measurements, genetic perturbation, or *in vivo* validation were lacking.

## 3 The T6SS as a multiscale regulator of host metabolism

### 3.1 A concise mechanistic framework for effector delivery

The canonical T6SS is built from a membrane complex, a cytoplasmic baseplate, a hemolysin-coregulated protein (Hcp) tube, a valine-glycine repeat protein G (VgrG)-proline-alanine-alanine-arginine (PAAR) puncturing spike, and a contractile TssB-TssC sheath. Assembly and firing are coordinated processes in which the extended sheath stores mechanical energy and then contracts to propel the tube, spike, and associated cargo across the bacterial envelope. The AAA+ (ATPases associated with diverse cellular activities) ATPase ClpV subsequently disassembles the contracted sheath and recycles its subunits (Basler, 2015; Kudryashev et al., 2015; Shneider et al., 2013; Vettiger et al., 2017). Effectors may be carried within Hcp, attached to VgrG or PAAR proteins, or fused to structural components; accessory proteins and loading domains further diversify cargo specificity (Colautti et al., 2024; Dyrma et al., 2025).

The delivery architecture described above has two direct consequences for host metabolism. First, T6SS firing is energetically costly and is therefore tightly regulated by environmental cues, quorum sensing, stress responses, and cargo availability (Basler, 2015; Dyrma et al., 2025; Storey et al., 2020). Second, the apparatus can deliver enzymes directly into a defined cellular compartment, creating spatially restricted perturbations that may be missed by bulk measurements. A phospholipase targeted to the ER, for example, can alter local lipid integrity and protein-folding stress without producing the same signature as generalized membrane damage (Jiang et al., 2016). Similarly, a metal-binding micropeptide can suppress an immune sensor by changing cofactor availability without globally depleting cellular metal stores (Zhu et al., 2021).

### 3.2 Three levels of evidence for T6SS-associated metabolic reprogramming

We propose a three-tier framework for evaluating metabolic claims. Tier 1 comprises direct biochemical or metabolic effects, such as cleavage of host phospholipids, sequestration of a metal cofactor, disruption of nutrient uptake, or changes demonstrated by metabolomics, isotope tracing, adenosine triphosphate (ATP) measurements, or extracellular-flux analysis (Jang et al., 2018). Tier 2 comprises organelle and signaling changes with plausible metabolic consequences, including mitochondrial fragmentation, ER stress, calcium transfer, reactive oxygen species (ROS) production, phosphoinositide 3-kinase (PI3K)-Akt activation, and altered autophagy (Jiang et al., 2016; Sá-Pessoa et al., 2023; Storey et al., 2020; Wettstadt et al., 2019). These observations are mechanistically important but should not automatically be described as metabolic reprogramming unless metabolic flux or metabolite abundance has been measured. Tier 3 comprises ecological effects in which T6SS-mediated competition changes community composition, nutrient release, or metabolite-producing capacity (Borgeaud et al., 2015; Lin et al., 2021; Ringel et al., 2017; Sana et al., 2017). Such effects may influence systemic host metabolism but require causal testing that separates T6SS activity from other strain-level differences (Table 1).

**TABLE 1:** Representative T6SS-associated mechanisms at the interface of metabolism and immunity.

System or effector	Primary target or context	Metabolic/ immunometabolic axis	Reported outcome	Evidence level for the stated metabolic claim
*P. aeruginosa* TplE	ER membrane; PGAP1-like targeting	Phospholipid integrity, IRE1alpha-XBP1 UPR, autophagy	ER stress and increased autophagic flux (Jiang et al., 2016)	Tier 1 (direct ER phospholipid injury); downstream host metabolic flux unresolved
*K. pneumoniae* VgrG4	Mitofusin 2 and ER-mitochondrial contacts	Mitochondrial Ca2+, Drp1-dependent fission, ROS	NLRX1 activation and suppression of NF-kappaB signaling (Sá-Pessoa et al., 2023)	Tier 2 (organelle and redox evidence; limited flux analysis)
*P. aeruginosa* PldA/PldB	Akt signaling during epithelial-cell entry	PI3K-Akt and potential anabolic/glucose signaling	Membrane remodeling and bacterial internalization (Wettstadt et al., 2019)	Tier 2 (signaling evidence; metabolic flux unverified)
*Y. pseudotuberculosis* TkeA	Nuclear DNA	DNA-damage signaling, cGAS-STING-TNF, apoptosis	Caspase-8-dependent apoptosis (Song et al., 2025)	Tier 1 (direct biochemical substrate damage); downstream metabolism unresolved
*Y. pseudotuberculosis* TssS	Intracellular Mn2+ pool	Metal-cofactor availability for cGAS-STING	Suppression of innate DNA sensing (Zhu et al., 2021)	Tier 1 (direct biochemical/metal evidence)
*E. tarda* EvpP	Ca2+-dependent MAPK-JNK signaling	Ion-sensitive inflammasome regulation	Inhibition of NLRP3 assembly and IL-1beta release (Chen et al., 2017)	Tier 2 (signaling and immune evidence)
*B. thailandensis* TseM	Extracellular manganese	Metal acquisition and oxidative-stress resistance	Improved manganese uptake and stress tolerance (Si et al., 2017)	Tier 1 (direct nutrient-acquisition evidence)
*Y. pseudotuberculosis* YezP	Extracellular zinc uptake network	Zinc acquisition through HmuR/ZnuABC	Support of bacterial metal homeostasis (Liu et al., 2023)	Tier 1 (direct nutrient-acquisition evidence)
*S. marcescens* Tfe2	Fungal nutrient uptake	Amino-acid metabolism and starvation response	Antifungal growth inhibition and cell death (Trunk et al., 2018)	Tier 1 (direct metabolic phenotype in target fungus)
Community-level T6SS activity	Competing bacteria in host-associated microbiota	Nutrient release, DNA uptake, community metabolite capacity	Competition, horizontal gene transfer, and altered community structure (Borgeaud et al., 2015; Ringel et al., 2017)	Tier 3 (ecological evidence; host metabolic causality often indirect)

Evidence levels follow the Tier 1 (direct biochemical or metabolic measurement), Tier 2 (organelle or signaling proxy), and Tier 3 (ecological inference) terminology defined in section “3.2 Three levels of evidence for T6SS-associated metabolic reprogramming.” For effectors with direct substrate-injury evidence, the Tier 1 designation refers to that direct biochemical effect, whereas evidence for downstream host metabolic reprogramming is indicated separately.

This hierarchy is intended to strengthen rather than narrow the field. It identifies where current mechanistic evidence is already compelling and where the next experimental step should be metabolic validation. It also helps prevent overinterpretation of surrogate endpoints. Increased ROS, for instance, may reflect mitochondrial respiration, nicotinamide adenine dinucleotide phosphate (NADPH) oxidase activity, metal imbalance, membrane injury, or impaired antioxidant defense (Sá-Pessoa et al., 2023; Si et al., 2017; Storey et al., 2020). Likewise, a change in mitochondrial morphology does not by itself establish a switch between oxidative phosphorylation and glycolysis.

## 4 Minimum evidence standards for T6SS-mediated metabolic reprogramming

A convincing claim of T6SS-mediated metabolic reprogramming should satisfy four criteria. First, dependence on the secretion system should be shown using a structural T6SS mutant and genetic complementation. Second, effector specificity should be established with an effector deletion and, when feasible, a catalytic-site mutant. Third, a direct metabolic endpoint should be measured, such as metabolite abundance, isotope incorporation, respiration, glycolytic flux, ATP/adenosine diphosphate (ADP), nicotinamide adenine dinucleotide (NAD+)/NADH, lipid species, or bioavailable metal. Fourth, the metabolic change should be linked to infection outcome by rescue, supplementation, inhibition, or genetic manipulation of the host pathway.

These standards are especially important when effectors also cause cell death. Metabolic measurements should be normalized to viable cell number and collected before extensive membrane rupture. Heat-killed bacteria, isogenic secretion-competent but effector-deficient strains, and conditioned-medium controls can separate contact-dependent intoxication from soluble inflammatory signals. Ectopic expression of effector constructs in host cells may complement–but should not substitute for–T6SS-dependent delivery, because these effectors are normally introduced into cells through the secretion apparatus rather than as free purified protein. Bacterial growth and viability must also be monitored because an apparent host-metabolic phenotype may reflect differences in inoculum fitness.

## 5 Organelle-centered immunometabolic reprogramming

### 5.1 ER lipid injury, unfolded-protein responses, and autophagy

The ER integrates lipid synthesis, calcium storage, protein folding, and communication with mitochondria. It is therefore a strategic target for pathogens seeking to alter both cellular viability and immune output. The *Pseudomonas aeruginosa* H2-T6SS effector TplE contains a PGAP1-like (post-GPI attachment to proteins 1) domain that directs the effector to the ER. Its phospholipase activity damages ER membranes and activates the inositol-requiring enzyme 1alpha (IRE1alpha)-X-box binding protein 1 (XBP1) arm of the unfolded-protein response (UPR), accompanied by increased autophagic flux (Jiang et al., 2016). This mechanism provides one of the clearest links between a T6SS-delivered enzyme and a host organelle with central metabolic functions.

The metabolic consequences of TplE activity are likely to extend beyond membrane injury. ER phospholipid integrity is required for lipoprotein synthesis, organelle contact sites, calcium transfer, and the adaptation of secretory and immune cells to stress. IRE1alpha-XBP1 signaling can reshape lipid biosynthesis and inflammatory gene expression (Hetz, 2012; Jiang et al., 2016), while autophagy can either eliminate damaged organelles and bacteria or provide nutrients that support intracellular persistence (Jiang et al., 2016). However, the published mechanism primarily establishes ER localization, UPR activation, and autophagy. Direct measurements of phospholipid species, ER-mitochondrial calcium exchange, cellular ATP, or carbon flux remain limited. Accordingly, TplE should be viewed as a strong candidate driver of immunometabolic reprogramming whose metabolic outputs still require systematic definition.

Several experimental strategies would clarify this pathway. Catalytically inactive TplE, T6SS-deficient bacteria, and complementation controls should be compared in primary macrophages and epithelial cells. Lipidomics could identify the phospholipid classes most affected (Jang et al., 2018), while ER-targeted calcium reporters and isotope-labeled fatty acids could determine whether membrane damage changes lipid synthesis or ER-mitochondrial exchange (Jang et al., 2018; Jiang et al., 2016). Parallel measurement of bacterial survival would establish whether the host stress response is protective, pathological, or exploited by the pathogen.

### 5.2 Mitochondrial calcium overload, dynamics, and redox imbalance

Mitochondria are central to cellular bioenergetics and innate immune signaling, making them an especially consequential target of T6SS effectors. *Klebsiella pneumoniae* VgrG4 contains a C-terminal toxic domain that interacts with mitofusin 2 at ER-mitochondrial contact sites. VgrG4 promotes calcium transfer to mitochondria, activates the fission regulator dynamin-related protein 1 (Drp1), and fragments the mitochondrial network. The accompanying activation of NLR family member X1 (NLRX1) and ROS production suppresses nuclear factor-kappaB (NF-kappaB) signaling through interference with the cullin-1 ubiquitin-ligase pathway, thereby favoring infection (Sá-Pessoa et al., 2023). *K. pneumoniae* also encodes the cognate immunity protein Sel1E, which protects the bacterium from self-intoxication, while T6SS-associated competition in this organism is linked to PhoPQ regulation and ROS-dependent conditions (Storey et al., 2020).

Within the evidence framework defined in section “3.2 Three levels of evidence for T6SS-associated metabolic reprogramming,” current VgrG4 data remain Tier 2 evidence: they rely on proxy markers–mitochondrial morphology, calcium imaging, ROS probes, and immunoblotting–rather than on direct bioenergetic measurements. This mechanism directly connects organelle contact sites, calcium, redox signaling, and immune suppression. Nevertheless, the term mitochondrial dysfunction encompasses several non-equivalent states. Fragmented mitochondria may show reduced respiratory reserve, increased proton leak, altered membrane potential, or compensatory glycolysis, but these outcomes cannot be inferred solely from morphology. Future studies should combine live-cell imaging with oxygen-consumption rate, extracellular acidification rate, ATP/ADP ratios, mitochondrial membrane potential, and targeted measurements of tricarboxylic-acid-cycle intermediates (Jang et al., 2018). Stable-isotope tracing could determine whether VgrG4 redirects glucose or glutamine carbon, while mitochondrial ROS probes should be interpreted alongside NADPH oxidase inhibition and antioxidant controls.

The cell type and metabolic baseline are also likely to determine outcome. Resting macrophages, activated macrophages, airway epithelial cells, and hepatocytes have distinct mitochondrial demands and antioxidant capacities. A VgrG4-mediated perturbation that suppresses inflammatory signaling in one cell type may trigger cell death or compensatory stress responses in another. This context dependence is directly relevant to comorbidities such as diabetes, obesity, and aging (Casqueiro et al., 2012; Fulop et al., 2018; Huttunen and Syrjänen, 2013), in which mitochondrial quality control and redox homeostasis are already altered.

### 5.3 PI3K-Akt signaling and cytoskeletal-metabolic coupling

*Pseudomonas aeruginosa* uses the H2-T6SS to deliver the phospholipase D effectors PldA and PldB, which engage Akt signaling and promote bacterial internalization (Wettstadt et al., 2019). The same secretion system deploys VgrG2b to interact with the gamma-tubulin ring complex and facilitate microtubule-dependent uptake into epithelial cells (Sana et al., 2015). These pathways are often discussed as cytoskeletal or invasion mechanisms, but PI3K-Akt is also a major regulator of glucose uptake, anabolic metabolism, mechanistic target of rapamycin (mTOR) signaling, and cell survival. T6SS-dependent activation of Akt could therefore create a transient metabolic state that supports membrane remodeling and pathogen entry.

Direct evidence for this metabolic extension remains incomplete. Studies have established Akt phosphorylation and cytoskeletal changes (Sana et al., 2015; Wettstadt et al., 2019), but generally have not quantified glucose uptake, glycolytic flux, mTOR activity, or lipid synthesis under conditions that isolate effector activity. The distinction matters because Akt may function locally at the membrane without producing sustained metabolic reprogramming. Time-resolved analysis is therefore essential: early signaling during bacterial entry should be separated from later responses to intracellular replication, tissue damage, and inflammatory mediators. Within the framework of section “3.2 Three levels of evidence for T6SS-associated metabolic reprogramming,” these Akt data remain Tier 2 (signaling) evidence: glycolytic proton-efflux rate, oxygen-consumption rate, and tricarboxylic-acid-cycle metabolite pools have not been quantified under effector-isolated conditions, and Seahorse extracellular-flux analysis, stable-isotope tracing, and quantitative metabolomics will be required to determine whether PldA and PldB truly redirect host nutrient utilization.

Other T6SS effectors illustrate how cytoskeletal perturbation can intersect with immune metabolism indirectly. *Vibrio cholerae* VgrG1 cross-links actin and changes intestinal macrophage distribution and motility, whereas *Burkholderia* TecA deamidates Rho GTPases and activates the Pyrin inflammasome (Aubert et al., 2016; Durand et al., 2012; Dutta et al., 2019; Ngo et al., 2025). *Burkholderia* T6SS-5 effectors promote host-cell fusion and cell-to-cell spread (Kostow and Welch, 2022; Lennings et al., 2019; Toesca et al., 2014). These activities can change phagocytosis, membrane trafficking, mechanotransduction, and inflammatory-cell recruitment, all of which alter local nutrient consumption. However, their metabolic consequences should be presented as testable hypotheses unless supported by direct flux or metabolite measurements.

## 6 Metabolically sensitive innate immune checkpoints

### 6.1 Bidirectional control of the cGAS-STING pathway

The cyclic GMP-AMP synthase (cGAS)-stimulator of interferon genes (STING) pathway (Ablasser and Chen, 2019) provides an instructive example of how the same secretion system can either activate or suppress an innate immune checkpoint through mechanistically distinct effectors. *Yersinia pseudotuberculosis* TkeA is a T6SS-delivered DNase that damages nuclear DNA. Cytosolic DNA fragments activate cGAS-STING signaling, but the downstream response is redirected toward tumor necrosis factor (TNF) and caspase-8-dependent apoptosis rather than a conventional protective interferon program (Song et al., 2025). In contrast, the T6SS micropeptide TssS suppresses cGAS-STING signaling by sequestering Mn2+, a cofactor that enhances cGAS sensitivity and STING activation (Wang et al., 2018; Zhu et al., 2021).

These findings demonstrate two forms of metabolic control. TkeA changes the abundance and localization of a signaling substrate, whereas TssS changes the availability of a metal cofactor. The latter represents direct biochemical manipulation of an immunometabolic resource and is therefore stronger Tier 1 evidence. The TkeA pathway is also metabolically relevant because DNA damage, apoptosis, NAD+ consumption, and mitochondrial stress are tightly coupled, but these downstream metabolic consequences have not yet been mapped in detail.

A priority for future work is to determine whether TkeA and TssS act independently, sequentially, or in different host niches. Quantification of intracellular free Mn2+, cyclic GMP-AMP (cGAMP), NAD+, ATP, and mitochondrial function in cells infected with single- and double-effector mutants would help resolve this question. Spatially resolved assays are particularly important because total cellular manganese may remain unchanged even when a locally accessible pool required for cGAS activation is depleted.

### 6.2 Inflammasomes, calcium, and cell-death metabolism

Inflammasomes are closely coupled to ion flux, mitochondrial stress, glycolysis, lipid signaling, and redox balance. T6SS effectors can suppress or activate these processes. *Edwardsiella tarda* EvpP inhibits a calcium-dependent mitogen-activated protein kinase (MAPK)-c-Jun N-terminal kinase (JNK) pathway, preventing apoptosis-associated speck-like protein containing a caspase activation and recruitment domain (ASC) oligomerization and NOD-, LRR- and pyrin domain-containing protein 3 (NLRP3) inflammasome activation (Chen et al., 2017). Conversely, *Vibrio proteolyticus* Tie1 and Tie2 activate NLRP3 after phagocytosis and induce gasdermin-dependent pyroptosis, with alternative execution through gasdermin E (GSDME) and caspase-3 when the canonical gasdermin D (GSDMD) pathway is impaired (Cohen et al., 2022). *Burkholderia* TecA activates the Pyrin inflammasome through Rho-GTPase deamidation (Aubert et al., 2016).

The *Francisella* pathogenicity island encodes a non-canonical, T6SS-like contractile secretion system–phylogenetically related to the T6SS but lacking several conserved core components–whose effectors mediate phagosomal membrane disruption, enabling cytosolic access and activation of the absent in melanoma 2 (AIM2) inflammasome (Belhocine and Monack, 2012; Cantlay et al., 2022). These observations establish that contractile secretion systems can control the timing and mode of inflammatory cell death. Metabolically, pyroptosis causes rapid loss of ion gradients and releases intracellular nutrients and danger signals, whereas apoptosis tends to preserve membrane integrity during early stages. The chosen death pathway can therefore influence bacterial dissemination, neutrophil recruitment, and resource availability within infected tissue. Importantly, such rapid, lethal lysis–for example, pyroptosis or necrosis with wholesale release of cellular contents–must be distinguished from active metabolic reprogramming of viable host cells: lysis terminates host metabolism, whereas reprogramming redirects it, and only the latter constitutes immunometabolic modulation in the sense used in this review.

Most current studies measure inflammasome assembly, cytokine release, and cell death rather than the metabolic state that precedes or follows these events. Integrating metabolomics with live-cell measurements of calcium, potassium, mitochondrial potential, and lactate release would clarify whether metabolic changes are upstream drivers, downstream consequences, or parallel responses to membrane injury. Such timing information is essential for identifying therapeutic windows.

## 7 Nutritional immunity, metal homeostasis, and redox control

### 7.1 T6SS-mediated access to zinc and manganese

**FIGURE 2:**
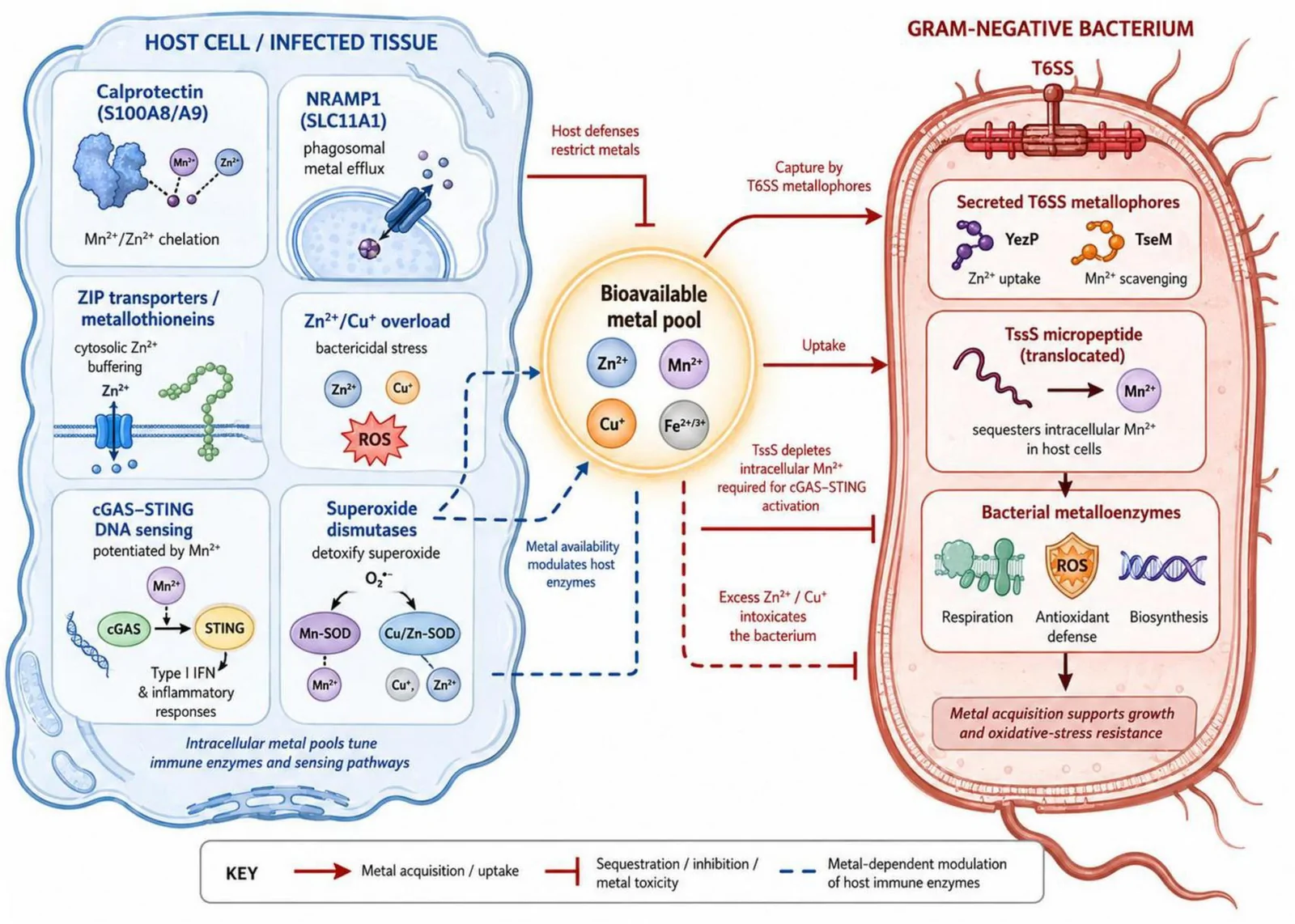
Host nutritional immunity and metal homeostasis at the interface with T6SS-mediated metal handling. Host and bacterial factors compete for a central bioavailable metal pool at the host–pathogen interface. On the host side, nutritional immunity restricts microbial access to essential transition metals: calprotectin (S100A8/A9) chelates Mn2+ and Zn2+ at infection foci, NRAMP1 (SLC11A1) extrudes metals from the phagosome, and ZIP-family transporters together with metallothioneins buffer cytosolic Zn2+, whereas Zn2+/Cu+ overload can also be deployed as a bactericidal stress. In parallel, host metal pools tune immune enzymes, including Mn2+-potentiated cGAS-STING DNA sensing and Mn- and Cu/Zn-dependent superoxide dismutases. Gram-negative pathogens counter these defenses through T6SS-associated metal handling: secreted metallophores (YezP and TseM) capture Zn2+ and Mn2+ for bacterial uptake, and the translocated micropeptide TssS sequesters the intracellular Mn2+ pool required for cGAS-STING activation. Acquired metals support bacterial metalloenzyme-dependent functions, including respiration, antioxidant defense, and biosynthesis. Solid arrows denote metal acquisition or uptake; blunt-ended lines denote sequestration, inhibition, or metal toxicity; dashed lines denote metal-dependent modulation of host immune enzymes. cGAS, cyclic GMP-AMP synthase; NRAMP1, natural resistance-associated macrophage protein 1; STING, stimulator of interferon genes; T6SS, type VI secretion system.

Hosts restrict microbial access to essential metals through nutritional immunity, while bacteria deploy high-affinity uptake systems and secreted metal-binding proteins to overcome this limitation. Several T6SS cargo proteins function as extracellular metallophores rather than contact-dependent toxins. *Y. pseudotuberculosis* YezP participates in zinc acquisition through interactions with HmuR and ZnuABC, whereas *Burkholderia thailandensis* TseM scavenges manganese and supports oxidative-stress resistance (Liu et al., 2023; Si et al., 2017). Together with the intracellular Mn2+-sequestering activity of TssS, these findings show that T6SS-associated proteins can manipulate metal availability in both extracellular and host-cell compartments. Nutritional immunity is, however, a two-sided battle for nutrient metals, and T6SS metallophores compete directly with host metal-restriction and metal-overload strategies (Figure 2).

On the host side, metal restriction and metal overload are both used as antimicrobial weapons (Hood and Skaar, 2012). The neutrophil- and epithelium-derived S100A8/A9 heterocomplex (calprotectin) chelates Mn2+ and Zn2+ at infection foci and within abscesses (Corbin et al., 2008; Hood and Skaar, 2012); the phagosomal transporter NRAMP1 (natural resistance-associated macrophage protein 1; SLC11A1) extrudes divalent metals from the phagosomal lumen (Cellier et al., 2007); and ZIP (Zrt/Irt-like protein)-family importers, metallothioneins, and metal-efflux systems reshape cytosolic pools, whereas excess Zn2+ and Cu+ are harnessed as bactericidal stressors (Djoko et al., 2015; Hood and Skaar, 2012). Host metal manipulation also tunes immune enzymes directly: Mn2+ potentiates cGAS-STING sensing of cytosolic DNA (Wang et al., 2018), and Mn- and Cu/Zn-dependent superoxide dismutases (SODs) depend on the same pools (Hood and Skaar, 2012). T6SS-secreted metallophores such as YezP and TseM, and translocated metal-binding micropeptides such as TssS, therefore compete directly with host chelators and transporters for nutrient metals (Figure 2). Genome-wide surveys have identified such secreted metal-binding proteins in diverse Gram-negative pathogens, suggesting that they are likely to be general participants in this contest (Sharma et al., 2017, 2018).

Metal acquisition has two simultaneous consequences. It supports microbial enzymes involved in respiration, antioxidant defense, and biosynthesis, and it changes the competitive environment for neighboring microbes. Manganese is particularly important for resistance to oxidative stress (Hood and Skaar, 2012; Si et al., 2017), whereas zinc is required for numerous enzymes but becomes toxic when present in excess (Djoko et al., 2015; Hood and Skaar, 2012). The effect of a T6SS metallophore therefore depends on local metal abundance, competing transporters, host sequestration proteins, and the oxidative state of the niche.

This area is well suited to quantitative analysis. Inductively coupled plasma mass spectrometry can measure total metal content, but genetically encoded or fluorescent sensors are needed to resolve bioavailable pools (Hood and Skaar, 2012). Mutants lacking the secreted metallophore, its receptor, or downstream transporter can separate capture from uptake (Liu et al., 2023; Si et al., 2017). Combining these tools with ROS measurements, bacterial fitness assays, and host cytokine profiling would link metal flow to both microbial metabolism and immune outcome.

### 7.2 Redox balance as both a target and a constraint

Type VI secretion system biology is deeply intertwined with redox stress. Some effectors promote ROS in host cells, while bacterial T6SS deployment and competition can be controlled by oxidative-stress pathways. In *K. pneumoniae*, T6SS-mediated competition is regulated by PhoPQ and influenced by ROS-dependent conditions (Storey et al., 2020). In *B. thailandensis*, manganese acquisition through TseM supports resistance to oxidative damage (Si et al., 2017). These relationships suggest that redox balance is not merely a consequence of effector intoxication; it is also a determinant of when T6SS activity is advantageous.

A useful conceptual distinction is between host-directed ROS and bacterial self-protection. Host ROS may contribute to microbial killing but can also damage tissue and suppress signaling when generated in specific compartments. Bacterial antioxidant systems protect the attacker during deployment and may determine whether T6SS-mediated competition succeeds in inflamed niches. Experimental designs should therefore quantify ROS in both organisms when possible and should avoid interpreting a single whole-cell fluorescent signal as evidence of a specific source.

The energetic cost of T6SS assembly adds another layer. Repeated sheath polymerization, firing, and recycling require substantial biosynthetic and ATP investment. Cargo-dependent assembly checkpoints may reduce waste by coupling apparatus completion to effector availability (Dyrma et al., 2025). Whether infection-associated nutrient limitation changes the balance between T6SS investment, growth, and stress tolerance remains largely unexplored. This question is particularly relevant in chronic infections, where bacteria experience spatially heterogeneous oxygen, carbon sources, and metal availability.

## 8 Cross-kingdom metabolic competition and microbiome remodeling

### 8.1 Antifungal activity as metabolic interference

T6SS-mediated competition extends to fungi that share mucosal, environmental, and biofilm niches with bacteria. *Serratia marcescens* delivers the antifungal effectors Tfe1 and Tfe2. Tfe1 depolarizes the fungal plasma membrane, whereas Tfe2 disrupts nutrient uptake and amino-acid metabolism, producing a starvation-like response (Trunk et al., 2018). Tfe2 therefore provides a clear example in which metabolic disruption is central to the toxic mechanism rather than a secondary consequence of generalized lysis.

Other antifungal effectors primarily damage structural or genetic targets. *Acidovorax citrulli* TseN and *Acinetobacter baumannii* TafE localize to fungal nuclei and degrade DNA, while a broad-spectrum antifungal effector has been proposed as a determinant of bacterial-fungal competition (Luo et al., 2023; Pei et al., 2022; Yan et al., 2025). *Y. pseudotuberculosis* can also sense the fungal quorum signal tyrosol and activate a T6SS-associated chitinase response in the gut (Zhu et al., 2026). These mechanisms illustrate reciprocal chemical surveillance: fungal metabolites regulate bacterial weapon deployment, and bacterial effectors then alter fungal nutrient access, membrane potential, or genome integrity.

For host-pathogen interactions, the key unanswered question is whether these cross-kingdom effects alter host metabolite exposure or colonization resistance. Reducing *Candida* abundance, for example, could change lactate, short-chain fatty acids, oxygen consumption, and epithelial signaling. Such community-level consequences are plausible but require gnotobiotic or defined-consortium models that compare wild-type bacteria with T6SS-deficient mutants while controlling for bacterial load.

### 8.2 Diet, circadian timing, and microbiome-derived metabolites

Diet supplies the substrates that shape both microbial competition and host metabolism. In *V. cholerae*, dietary composition can alter T6SS regulation and competitive outcomes with the gut microbiota, providing direct evidence that nutrient context changes the ecological value of the system (Liu et al., 2025). This observation is important because T6SS activity may have opposite consequences under different diets: it can facilitate niche clearance when competition is intense, yet its energetic cost may be disadvantageous when nutrients or regulatory signals suppress deployment.

Circadian feeding patterns and dietary macronutrients also alter gut-community structure and metabolite production. Microbiome-derived bile acids can signal through host receptors such as the G protein-coupled bile acid receptor TGR5 and influence vascular and immune physiology, while diet-associated dysbiosis has been linked to cardiometabolic disease (Feng et al., 2026; Qi et al., 2025). However, current evidence does not establish a direct causal chain from a specific T6SS effector to altered bile acids and then to atherosclerosis. T6SS abundance or expression may correlate with broader virulence and ecological shifts, but causal attribution requires genetic perturbation of the T6SS within otherwise matched strains.

Future studies should combine time-restricted feeding, defined diets, and isogenic T6SS mutants with longitudinal metagenomics, metatranscriptomics, and metabolomics. Sampling time must be standardized because both microbial gene expression and host metabolites display circadian variation. Candidate metabolites should then be tested by supplementation, receptor antagonism, or microbial reconstitution rather than inferred solely from correlation networks.

### 8.3 Commensal T6SSs, colonization resistance, and gene flow

T6SS activity is not synonymous with pathogenesis. Commensal and symbiotic bacteria use related systems to structure their communities and influence host responses. In the honey-bee gut, *Snodgrassella alvi* T6SSs promote strain competition and modulate host antimicrobial-peptide expression (Motta et al., 2024). In the oral cavity, *Aggregatibacter aphrophilus* uses T6SS effectors to antagonize a periodontal pathogen and reduce inflammatory burden in epithelial-cell models (Bao et al., 2025). These examples suggest that the host outcome of T6SS activity depends on which organism carries the system, which competitors are removed, and how the remaining community contributes to metabolism and barrier function.

T6SS-mediated lysis also releases DNA and nutrients. In naturally competent bacteria such as *V. cholerae* and *Acinetobacter baylyi*, killing can be coordinated with DNA uptake, increasing horizontal gene transfer (Borgeaud et al., 2015; Ringel et al., 2017). This process can accelerate acquisition of antimicrobial-resistance or metabolic loci and thereby alter future community behavior. Its relevance to immunometabolism is indirect but important: resistance acquisition changes antibiotic exposure and community composition, while newly acquired metabolic genes may expand substrate use in the infected niche.

These ecological effects reinforce the need to evaluate T6SSs at the strain and community levels. A host-beneficial T6SS in one consortium may be harmful in another. Therapeutic manipulation should therefore prioritize defined targeting and ecological monitoring rather than global activation or inhibition of all T6SS activity.

## 9 Metabolic comorbidities as potential determinants of T6SS-mediated responses

The Research Topic emphasis on diabetes, obesity, and aging is highly relevant to T6SS biology, but direct mechanistic evidence remains limited. This section is therefore explicitly hypothesis-generating–a future outlook rather than a summary of established mechanisms–and the hypotheses below are anchored where possible in documented metabolic alterations of the comorbid host. These conditions share features that could modify effector action, including altered glucose and lipid availability, chronic low-grade inflammation, impaired mitochondrial quality control, endothelial dysfunction, and changes in metal homeostasis. The appropriate conclusion is therefore not that T6SSs are established mediators of comorbidity-associated infection, but that comorbid metabolic states may change the threshold and outcome of T6SS-dependent perturbations. For instance, elevated baseline glucose and altered lipid profiles in diabetic or obese tissue microenvironments could plausibly influence Gram-negative envelope composition and PhoPQ two-component regulation–a known controller of T6SS deployment (Storey et al., 2020)–and may raise baseline organelle stress (Casqueiro et al., 2012; Huttunen and Syrjänen, 2013); either effect could change host susceptibility to T6SS intoxication, although direct evidence in comorbid hosts is lacking.

Diabetes provides several testable possibilities. Hyperglycemia could modify bacterial carbon use and biofilm formation and might thereby indirectly influence secretion-system activity (Casqueiro et al., 2012; Storey et al., 2020), although direct regulation of the T6SS by diabetic conditions has not been demonstrated. In parallel, host cells may have impaired mitochondrial reserve and redox buffering. VgrG4-induced mitochondrial calcium stress or TplE-induced ER stress could therefore be amplified in diabetic cells even if effector delivery is unchanged. Conversely, a high-glucose environment might reduce the relative value of T6SS-mediated nutrient acquisition. These alternatives can be tested using primary cells from diabetic models, glucose-controlled media that avoid osmotic confounding, and infection with isogenic T6SS and effector mutants.

Obesity changes circulating lipids, adipokines, bile acids, and gut-community structure (Huttunen and Syrjänen, 2013). Phospholipase effectors may encounter different membrane compositions, and microbiome-level T6SS competition may produce different metabolite outputs under high-fat diets. Aging adds reduced autophagy, impaired mitophagy, altered inflammasome responses, and immunosenescence (Fulop et al., 2018). In aged hosts, the same organelle injury could lead to prolonged inflammation or defective bacterial clearance rather than the response observed in young animals. Direct comparison across age and metabolic status should therefore be incorporated early, rather than added only after a mechanism has been established in immortalized cells.

Clinically relevant models must also consider tissue context. Pulmonary, intestinal, urinary, and bloodstream infections differ in oxygen tension, nutrient supply, resident microbiota, and immune-cell composition. A T6SS mechanism identified in a macrophage monoculture may not dominate in a tissue where epithelial cells, neutrophils, and competing microbes determine the outcome. Multicellular organoids, air-liquid-interface cultures, and defined microbial consortia can bridge this gap before costly animal studies.

## 10 Therapeutic implications

### 10.1 Host-directed metabolic interventions

Host-directed therapies aim to improve antimicrobial defense or limit tissue damage by modifying host pathways rather than directly killing the pathogen. T6SS-associated mechanisms suggest several candidate targets: ER-stress and autophagy pathways for TplE-like effectors; mitochondrial calcium handling, Drp1 activity, or antioxidant responses for VgrG4; metal availability for TssS-mediated suppression of cGAS-STING; and inflammasome or gasdermin pathways for effectors that alter pyroptosis. These targets are attractive because they may remain effective against antibiotic-resistant organisms.

The principal challenge is pathway directionality. Autophagy, ROS, cGAS-STING, and inflammasomes can be protective or pathological depending on timing and tissue. Broad inhibition may reduce tissue injury but also impair bacterial clearance. Therapeutic studies should therefore define whether the desired effect is restoration of an inhibited host response, prevention of excessive inflammation, or protection of organelle function. Interventions should be tested at multiple times after infection and should include bacterial burden, tissue pathology, and host survival rather than cytokines alone.

Safety and feasibility constraints are equally important. Many candidate host targets sit at the core of cellular homeostasis: Drp1-dependent mitochondrial fission is essential for cardiac and neural development and function (Ikeda et al., 2015; Ishihara et al., 2009) and can be inhibited pharmacologically (Cassidy-Stone et al., 2008), while IRE1alpha-driven unfolded-protein signaling sustains protein-folding homeostasis across tissues (Hetz, 2012). Broad, systemic inhibition of these central organelle-stress and mitochondrial-dynamics pathways therefore carries a high risk of severe off-target toxicity in uninfected organs. Host-directed metabolic strategies should therefore be developed together with compartment-specific delivery (for example, inhaled formulations for pulmonary infection), transient and locally restricted therapeutic windows, and biomarker-guided patient stratification. Without these prerequisites, systemic modulation of central organelle-stress or mitochondrial-dynamics pathways is unlikely to be clinically tolerable.

Metal-targeted therapy requires particular caution. Supplementing manganese could theoretically restore cGAS activity after TssS-mediated sequestration but may also enhance bacterial antioxidant defenses. Conversely, systemic chelation could restrict microbial growth while weakening metal-dependent host enzymes. Local delivery, compartment-selective agents, or targeting the effector-metal interaction may provide safer alternatives.

### 10.2 Pathogen-directed anti-virulence strategies

A complementary approach is to inhibit the T6SS apparatus, effector loading, or effector catalytic activity. Because T6SS inhibition does not necessarily block bacterial growth, it may impose less direct selection than bactericidal therapy and could restore susceptibility to immune clearance or microbial competition. Potential targets include membrane-complex assembly, sheath dynamics, ClpV-dependent recycling, VgrG-PAAR cargo loading, and effector active sites. The increasing recognition of accessory loading motifs, including secretion determinants associated with the marker for type VI secretion (MIX) domain, may support cargo-specific inhibition (Fridman et al., 2022).

Anti-virulence therapy also has limitations. T6SSs can support colonization resistance when carried by commensals, and disabling a pathogen T6SS may have little effect if disease is driven by another secretion system or toxin. Inhibitors must therefore be evaluated in community models and in infections where the T6SS is demonstrably active. Biomarkers such as Hcp secretion, sheath imaging, effector-specific host signatures, or transcriptional reporters could help select responsive infections.

### 10.3 Precision microbiome engineering and combination therapy

Engineered bacteria offer a third strategy. A customizable antibacterial T6SS platform has been constructed in *Vibrio natriegens*, illustrating that effector payloads and regulatory circuits can be transferred into a tractable chassis (Jana et al., 2021). In principle, engineered commensals could remove a defined pathobiont, deliver an antifungal effector, or express immunity proteins that resist pathogen attack. This ecological precision contrasts with broad-spectrum antibiotics, which can cause prolonged collateral damage to commensal communities and increase susceptibility to secondary infection (Khanna and Pardi, 2016; Maier et al., 2021).

Safety and containment are central. Engineered T6SSs should be inducible, target-restricted, and unable to transfer cargo genes to resident microbes. The metabolic output of the edited community must also be monitored because successful pathogen removal does not guarantee restoration of beneficial metabolites. Combination therapy may be more realistic than a stand-alone T6SS intervention: an anti-virulence agent could reduce immune suppression, a conventional antimicrobial could lower burden, and a metabolically informed HDT could protect tissue or restore immune-cell function.

Therapeutic development should therefore be guided by mechanism-based stratification. A VgrG4-dominant infection may be best addressed by preserving mitochondrial function, whereas a TssS-dominant mechanism may require restoration of metal-dependent innate sensing. A microbiome-engineering approach may be preferable when T6SS-mediated competition, rather than direct host intoxication, is the major driver.

## 11 Models, technologies, and outstanding questions

### 11.1 Models and technologies

Extracellular-flux analysis provides rapid assessment of respiration and glycolysis but should be complemented by targeted metabolomics and isotope tracing. Stable-isotope-labeled glucose, glutamine, fatty acids, or acetate can identify substrate redistribution, while lipidomics can define ER and mitochondrial membrane changes. Genetically encoded sensors are useful for compartment-specific ATP, calcium, redox state, and metal availability. Spatial metabolomics and imaging mass spectrometry may be particularly valuable in infected tissues where T6SS activity is localized.

Single-cell RNA sequencing can reveal heterogeneous immune states, but transcript-based pathway scores are not direct measurements of metabolism. Integration with single-cell assay for transposase-accessible chromatin (ATAC) sequencing, protein measurements, isotope tracing, or spatial metabolomics is needed to distinguish transcriptional potential from actual flux. Multi-omics studies should be anchored by isogenic genetics and predefined causal models rather than relying only on correlation networks.

Model selection should follow the proposed mechanism. Primary macrophages and epithelial cells are appropriate for organelle and innate-signaling studies; organoids and air-liquid-interface cultures are better for barrier metabolism; gnotobiotic animals and defined consortia are required for community-level claims. Diabetes, obesity, or aging models should be included when the hypothesis concerns metabolic susceptibility, but they should not substitute for identifying the effector-dependent pathway.

### 11.2 Outstanding questions

Several questions can now be addressed experimentally: (1) Which T6SS effectors directly alter host metabolic enzymes or metabolites rather than acting through organelle injury? (2) Do TplE and VgrG4 cause reproducible changes in carbon flux, lipid composition, or respiratory reserve in primary cells? (3) How are metal acquisition, metal sequestration, and oxidative-stress resistance coordinated within the same infection niche? (4) Which dietary or circadian conditions increase the ecological benefit of T6SS deployment? (5) Do diabetes, obesity, or aging amplify specific effector phenotypes, and can the vulnerability be therapeutically reversed? (6) Can T6SS-dependent host or metabolite signatures identify patients most likely to benefit from HDTs or anti-virulence therapy?

## 12 Conclusion

Throughout this review, we have distinguished active metabolic reprogramming of viable host cells from generalized, lethal cell lysis–for example, rapid pyroptosis or necrosis that simply releases cellular contents–because only the former constitutes immunometabolic modulation. Its effectors and associated cargo proteins act at metabolic control points that include ER lipid homeostasis, mitochondrial calcium and redox signaling, metal availability, nutrient uptake, and microbial-community resource allocation. The strongest current evidence concerns direct phospholipid injury, manganese and zinc handling, fungal nutrient starvation, and organelle-centered immune modulation. Other proposed systemic metabolic consequences remain plausible but incompletely proven.

An immunometabolic framework brings two advantages. It connects diverse T6SS mechanisms through shared host processes, and it defines the measurements needed to move from descriptive cell injury to causal metabolic biology. Direct flux analysis, compartment-specific sensors, isogenic genetics, and physiologically relevant models will be essential. Equally important, future studies should distinguish host-directed therapy from pathogen-directed anti-virulence and microbiome-engineering approaches, because these strategies act at different levels and carry different risks.

By linking effector biochemistry to cellular metabolism, immune function, and community ecology, the field can identify when the T6SS is a driver of disease, a marker of competitive fitness, or a potentially beneficial component of colonization resistance. This distinction will determine whether T6SS-associated pathways can be translated into precise interventions for complex infections.
